# Exploring Pain on Social Media: Observational Study on Perceptions and Discussions of Chronic Pain Conditions

**DOI:** 10.2196/67473

**Published:** 2025-09-16

**Authors:** Teresa Valades, Cesar I Fernandez-Lazaro, Francisco Lara-Abelenda, Maria Montero-Torres, Ines Cuberta Gonzalez, Miguel A Ortega, Melchor Alvarez-Mon Soto, Miguel Angel Alvarez-Mon

**Affiliations:** 1 Department of Medicine and Medical Specialties, Faculty of Medicine and Health Sciences, University of Alcala, C/ 19, Av de Madrid, Km 33,600, Alcalá de Henares, Madrid, 28871, Spain, 34 916 262626; 2 Ramon y Cajal Institute of Sanitary Research (IRYCIS), Ramon y Cajal Hospital, Madrid, Spain; 3 Department of Anesthesiology, Critical Care, and Pain Therapy, University Hospital of Torrejon, Torrejon de Ardoz, Spain; 4 Department of Preventive Medicine and Public Health, School of Medicine, University of Navarra, Pamplona, Spain; 5 IdiSNA, Navarra Institute for Health Research, Pamplona, Spain; 6 Department of Signal Theory and Communications, Telematics and Computing Systems, University of Rey Juan Carlos, Madrid, Spain; 7 Immune System Diseases-Rheumatology and Internal Medicine Service, Center for Biomedical Research in Hepatic and Digestive Diseases Network, University Hospital Principe de Asturias, Alcala de Henares, Madrid, Spain; 8 Department of Psychiatry and Mental Health, Infanta Leonor University Hospital, Madrid, Spain; 9 CIBERSAM-ISCIII (Biomedical Research Networking Centre in Mental Health), Madrid, Spain

**Keywords:** headache, paraplegia, multiple sclerosis, neuralgia, chronic pain, infodemiology, social media, fibromyalgia

## Abstract

**Background:**

Chronic pain, affecting 30.3% of the global population, constitutes a major public health and social challenge. It is associated with disability, emotional distress, and diminished quality of life. Conditions, such as fibromyalgia, headache, paraplegia, neuropathy, and multiple sclerosis are characterized by persistent pain and limited social and medical understanding. This contributes to patient isolation and increases mental health burden. In recent years, social media, particularly X (formerly Twitter), has emerged as a key space for analyzing health-related perceptions and experiences. Its massive use, spontaneity, and broad reach have made these platforms a valuable source for infodemiological research.

**Objective:**

This study aims to analyze posts on X concerning fibromyalgia, headache, paraplegia, neuropathy, and multiple sclerosis, as well as characterize the profile of users involved in these conversations, identify prevalent topics, measure public perception, evaluate treatment efficacy, and detect discussions related to the most frequent nonmedical issues.

**Methods:**

A total of 72,874 tweets in English and Spanish containing the selected keywords were collected between 2018 and 2022. A manual review of 2500 tweets was conducted, and the larger subset was automatically classified using natural language processing methods based on the BERTweet model, previously fine-tuned for content analysis on social media platforms. Subsequently, tweets related to chronic pain conditions were analyzed to examine user types, disease origin, and both medical and nonmedical content.

**Results:**

Of the total tweets collected, 55,451 (76.1%) were classifiable. The most active users were health care professionals and institutions. The primary perceived etiology was pharmacological, and higher treatment efficacy was noted in neuropathy, paraplegia, and multiple sclerosis. Regarding nonmedical content, there were more tweets related to the definition and understanding of the disease.

**Conclusions:**

Social media platforms, such as X, are playing a crucial role in the dissemination of information on chronic pain. Discussions largely focus on the available treatments and the need to enhance public education, using these platforms to correct misconceptions and provide better support to patients.

## Introduction

Chronic pain is defined as “an unpleasant sensory and emotional experience associated with actual or potential tissue damage or described in terms of such damage” (International Association for the Study of Pain) [1]. This type of pain is considered chronic when it persists beyond the normal duration of time, typically for more than 6 months [2]. Chronic pain has a global prevalence of 30.3%, representing a significant impact on global health [3], with important implications both on a personal and social level, leading to substantial loss of income, productivity, and quality-adjusted life years worldwide [4]. Moreover, individuals living with chronic pain often report feeling misunderstood by both their social environment and health care professionals [4]. This lack of understanding can exacerbate the emotional impact of pain, leading to feelings of isolation and frustration [5-7]. The absence of an adequate response from health care institutions and professionals may aggravate these feelings, causing patients to feel neglected and hopeless [5-8].

There are several diseases with neurological and musculoskeletal involvement that manifest with chronic pain, such as fibromyalgia, headache, paraplegia, neuropathy, and multiple sclerosis. These pathologies are a leading cause of disability and are associated with high morbidity rates, significantly impacting individuals’ quality of life [9]. These pain conditions contribute to mental health issues, such as anxiety and depression, increasing the global disease burden and indirect mortality due to factors such as suicide and accidents resulting from temporary disability [9,10].

Social media has attracted the attention of millions of users worldwide due to the possibility of rapid communication, access to a vast amount of information, and its wide dissemination [11,12]. According to studies, more than 55% of the global population used social media in 2022 [13]. Therefore, in recent years, medical research has focused on analyzing social media posts to understand diseases and their therapeutic processes better [14]. In addition, social media allows individuals to create and share content in a more informal and spontaneous environment, unlike traditional media, where users are passive consumers [11,15-18]. X (formerly Twitter) is one of the most popular and widely used platforms, considered an effective communication channel [19] and the most used in health research, with content analysis as the main focus [18,20,21]. For example, several studies have demonstrated a correlation between content published on these platforms and real-world clinical events, such as suicide rates [22-24], influenza outbreaks [25], or the misuse of medications and psychoactive substances [26-28].

Only a few studies have used social media to evaluate information related to patients with chronic pain [18,29-36]. In our study, we analyze the natural language used in posts extracted from X regarding 5 chronic pain diseases with the following objectives: (1) conduct a quantitative analysis of posts on X from 2018 to 2022 concerning headache, fibromyalgia, paraplegia, neuropathy, and multiple sclerosis, and determine which disease is most frequently discussed and which generates the greatest interest among users; (2) characterize the user profile that most actively participates in these discussions; (3) identify the etiopathogenesis of these diseases as attributed by X users; (4) analyze public perceptions regarding the treatment of these diseases; and (5) identify the most frequently discussed nonmedical topics.

## Methods

### Data Collection

This observational study used both quantitative and qualitative approaches and focused on the content of tweets related to a group of chronic pain–associated conditions, as published on the social media platform X. The following conditions were selected: headache, fibromyalgia, paraplegia, neuropathy, and multiple sclerosis, as they are frequent reasons for consultation in chronic pain clinics and are associated with long-term disability and reduced quality of life [37-42]. All tweets referring to these conditions were collected according to the following inclusion criteria: (1) tweets had to be publicly available (ie, from open accounts); (2) they had to include one or more of the following keywords (mentioned in the tweet text): “fibromyalgia,” “headache,” “migraine,” “multiple sclerosis,” “polyneuropathy,” “neuropathy,” “neuralgia,” “paraplegia,” “tetraplegia,” and their Spanish equivalents; (3) they had to be posted between January 2018 and December 2022, a broad time frame that allows for the capture of sustained and meaningful social media discussions on the topic; and (4) Tweets had to be written in English or Spanish, ensuring the representativeness of publications from different regions. Additional metadata were also collected for each tweet, including the number of retweets and likes, as indicators of user engagement and interest in the content [35,43].

Tweet Binder was the tool used to search for and collect the tweets included in this study, which we have used extensively in prior research [35,44,45], and which is capable of accessing 100% of public tweets. Moreover, Tweet Binder does not retrieve tweets from accounts it identifies as potential bots. It applies a hybrid approach that combines Botometer and graph-based bot detection to achieve this. Combining these methods enables more accurate detection. Botometer identifies anomalies at the individual account level while graph-based bot detection analyzes coordinated content propagation at the network level. To filter tweets by language during the search, we used the lang operator provided by the Twitter API v2. For example, lang:en was used to select tweets written in English and lang:es for those in Spanish.

### Content Analysis Process

**Figure 1.**
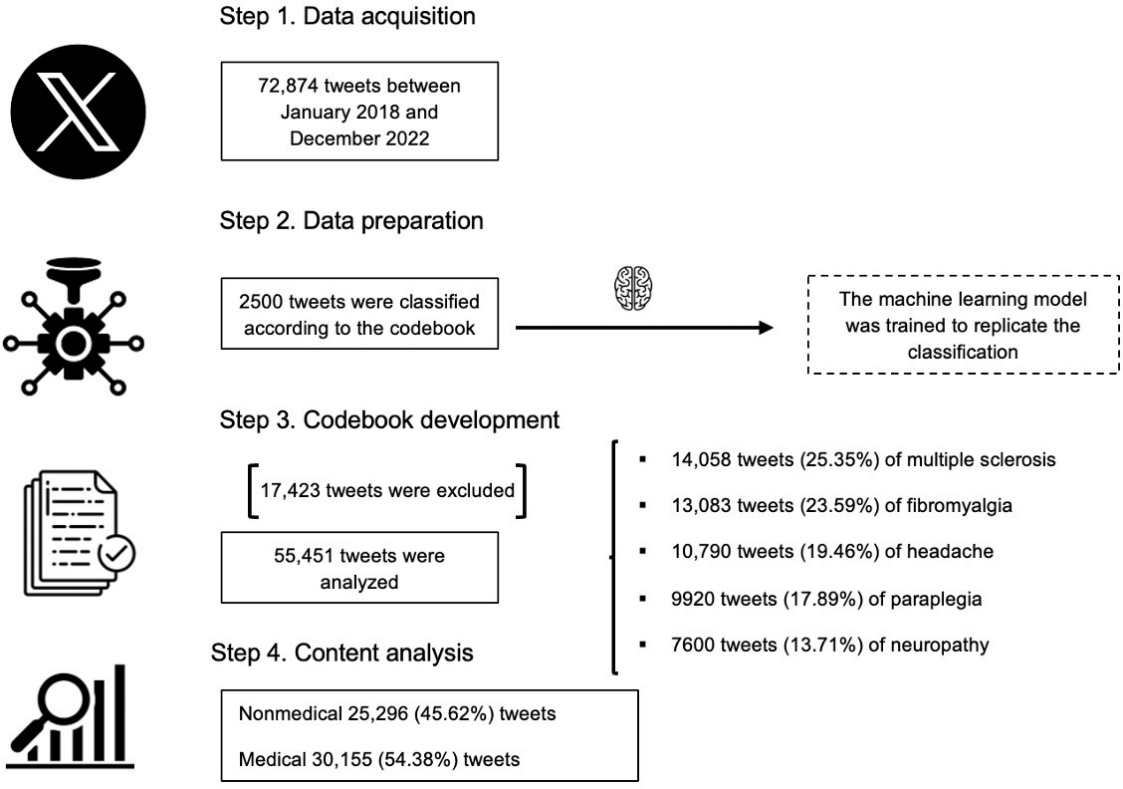
Flowchart of the study design.

Content analysis was conducted using a deductive approach, grounded in a solid theoretical framework derived from a prior review of the scientific literature. A total of 72,874 tweets were collected (Figure 1). A codebook (Multimedia Appendix 1) was developed to guide the analysis, and a subset of 2500 tweets was manually classified. Although the thematic categories were initially defined in the codebook, some flexibility was allowed during the analysis to refine the classification scheme.

Tweets were deemed nonclassifiable if their content was unrelated to the study objectives or if it lacked sufficient information to yield relevant insights. First, we analyzed the type of user. Users were categorized as patients, acquaintances of patients, health care professionals, or health care institutions. This categorization was based on the use of personal pronouns (useful for distinguishing patients from acquaintances), information available in the user’s Twitter profile (to identify professionals and institutions), or the tweet content itself (eg, the author self-identifying as a patient, relative, or professional). Next, we distinguished between medical and nonmedical content. Tweets were classified as “medical” when they referred to the origin or cause of the disease or its treatment. Regarding disease origin or cause, we further classified the tweets based on whether they mentioned a previous infection, vaccination, stress, or drug administration. For medical content, we also assessed whether the treatment described was perceived as effective or ineffective in managing chronic pain. Nonmedical content was categorized into three main themes: (1) knowledge or understanding of the disease, (2) commercial or advertising-related issues, and (3) legal concerns. Classification criteria and tweet examples are provided in Table 1.

**Table 1.** Category, definitions, and classification examples.

Category	Definition	Example
User type (refers to the individual or organization that posts or shares the tweet)*.*		
Patients	Personal experience with the disease	“Today I’m feeling a bit down. I’ve been struggling with a migraine since yesterday and ran out of medication.”
Patients’ acquaintances	Experience of a relative or friend with the disease.	“We’re raising money for my dear cousin who has a very aggressive and progressive form of multiple sclerosis.”
Health care professionals and institutions	Health care professional or institutional account.	“A 5% lidocaine patch is recommended for the relief of pain associated with neuropathy.”
Cause (probable etiology of the disease).		
Vaccine or previous infection	Previous infections or postvaccination processes.	“A moderate correlation has been demonstrated between HHV-6 infection and nerve fiber damage in chronic fibromyalgia.”
Stress	Physical or emotional.	“Chronic stress appears to be affecting many individuals suffering from migraines.”
Medications	As a trigger for the disease.	“Systematic review of cases: Linezolid-associated neuropathy.”
Treatment Efficacy (whether the treatment is perceived as effective or not for chronic pain)*.*		
Effective for chronic pain		“Spinal cord stimulation helps men with paraplegia walk again: Medical News Today.”
Not effective for chronic pain		“Focusing fibromyalgia treatment solely on inflammation is unlikely to result in optimal improvements in quality of life.”
Nonmedical content		
Knowledge	Refers to general information about the diseases: definitions/theories/ diagnostic criteria/classification, etc	"Fibromyalgia causes widespread body pain, extreme fatigue, and cognitive dysfunction. Symptoms may lead to other complications such as depression and anxiety and can have a significant impact on daily life.”
Commercial or advertising	Refers to promotion of the disease or related topics.	“Donations greatly support multiple sclerosis research. Follow the benefit here. Donate to the cause and win prizes.”
Legal or judicial	Refers to legal, political, social, or police complaints or claims	“Cannabis regulation is a powerful therapeutic weapon against multiple sclerosis worth advocating for.”
Usernames and personal names were removed.		

### Machine Learning Classification

Manually analyzing large datasets composed of thousands of tweets is often impractical; therefore, machine learning appears as a crucial tool in data analysis, encompassing 3 primary methodologies, such as supervised, unsupervised, and semisupervised learning [46]. This study focuses on semisupervised learning, which integrates elements from both supervised and unsupervised techniques by using a combination of labeled and unlabeled data to develop a machine learning model that replicates expert evaluations for the classification of millions of tweets. After preprocessing—which included tweet normalization, expansion of negative contractions, removal of special characters and repeated text, and conversion of emojis to their textual equivalents—the tweets were translated into English to improve performance in certain machine learning applications [47]. The dataset, composed of 2500 manually labeled tweets, is then randomly divided into 2 subsets: 75% (1875 tweets) for training and 25% (625 tweets) for testing. The decision to label 2500 tweets was based on a review of similar approaches previously described in the scientific literature [48,49], aiming to ensure consistency with established methodologies. The BERTweet model was selected due to its extensive application in the literature [50,51] and its training specifically on English tweets similar to those we are evaluating. To ensure that these models accurately replicate expert analyses, fine-tuning was conducted with the support of techniques, such as easy data augmentation [52] to balance categories. The models were evaluated on the test set by comparing artificial intelligence–generated predictions with manually annotated labels. To ensure robust evaluation, the *F*_1_-score metric was used, yielding the following results: 0.76 for user type, 0.77 for cause, 0.87 for treatment efficacy, and 0.67 for nonmedical content. These results indicate that the model demonstrates strong and consistent performance, aligned with similar methodologies reported in the literature [53,54].

### Statistical Analysis

The analyses in this study were descriptive, as no formal hypotheses were defined. The primary outcome of the study was the number of tweets containing the study keywords during the study period. Secondary outcomes included the number of likes and retweets of the corresponding tweets, as well as the like-to-tweet ratio and retweet-to-tweet ratio. Subgroup analyses included disease type, user type, disease etiology, medical content (efficacy), and nonmedical content (knowledge, advertising, and legal content). Descriptive statistics, including frequencies, proportions, and ratios, were used to summarize the number of tweets, likes, and retweets. The like-to-tweet ratio was calculated by dividing the number of likes by the number of tweets while the retweet-to-tweet ratio was determined by dividing the number of retweets by the number of tweets. All statistical analyses were conducted using STATA (version 16; StataCorp LP).

### Ethical Considerations

This study received approval from the Research Ethics Committee of the University of Alcalá (Code CEI: CEID/2024/1/005) and adheres to the ethical research principles established in the Declaration of Helsinki. This research did not involve human participants directly nor include any human intervention, as it used publicly available tweets. However, special care has been taken not to disclose users’ names or any information that could reveal users’ identities in this report.

## Results

### Total Tweet Count

A total of 72,874 tweets were obtained. According to the codebook, 55,451 (76.1%) were classifiable while 17,423 (23.9%) tweets were excluded. The classifiable tweets represented the diseases in the following order: multiple sclerosis with 14,058 (25.35%) tweets, fibromyalgia with 13,083 (23.59%) tweets, headache with 10,790 (19.46%) tweets, paraplegia with 9920 (17.89%) tweets, and finally, neuropathy with 7600 (13.71%) tweets. Based on their content, of the 55,451 tweets, 30,155 (54.38%) were classified as “medical” and 25,296 (45.62%) as “non-medical” (Figure 1). Regarding user engagement with the content, headache stood out with a like-to-tweet ratio of mean 468.80 (SD 33147.94) and a retweet-to-tweet ratio of mean 76.76 (SD 523.88), followed by multiple sclerosis with a like-to-tweet ratio of mean 163.12 (SD 1912.52) and a retweet-to-tweet ratio of mean 47.97 (SD 486.72; Table 2).

**Table 2.** Number of tweets published and impact ratios by disease.

Category	Original tweets, n (%)	Ratio like-tweet, mean (SD)	Ratio retweet-tweet, mean (SD)
Multiple sclerosis	14,058 (25.35)	163.12 (1912.52)	47.97 (486.72)
Fibromyalgia	13,083 (23.59)	70.28 (409.09)	27.68 (96.94)
Headache	10,790 (19.46)	468.80 (33147.94)	76.76 (523.88)
Paraplegia	9920 (17.89)	10.37 (137.56)	4.65 (74.49)
Neuropathy	7600 (13.71)	13.03 (125.69)	4.19 (47.92)

### Most Active Specific User Group: Health Care Professionals and Institutions

**Figure 2.**
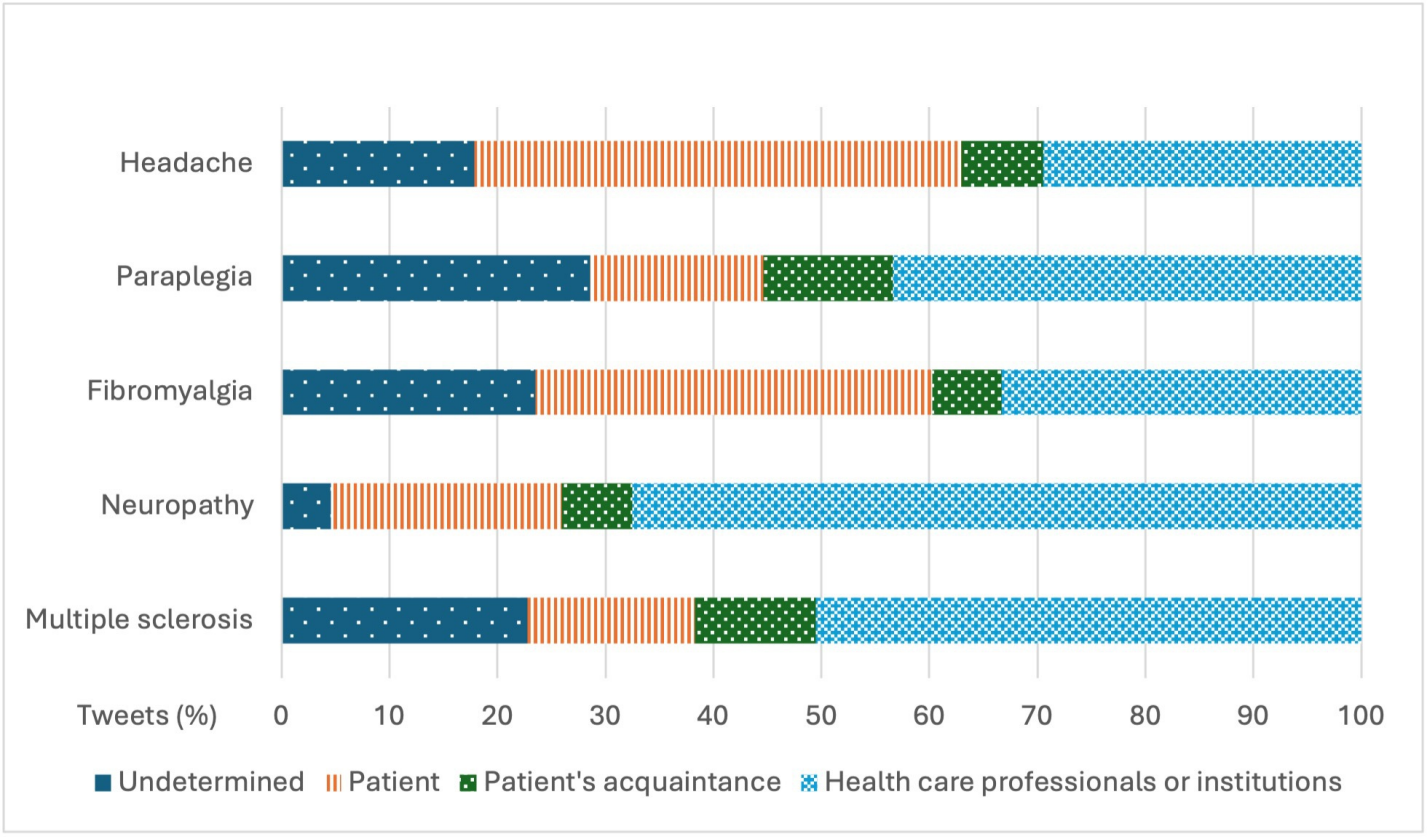
Percentage of tweets published for each disease by user type.

The users who published the most tweets related to chronic pain–related diseases were “health care professionals and institutions” with 24,080 (43.43%) tweets, followed by “patients” with 15,085 (27.2%) tweets and “patient acquaintances” with 4900 (8.84%) tweets. When comparing by disease (Figure 2), “patients” were the most active specific user group in tweets related to headache, with 4865/10,790 (45.09%) tweets and fibromyalgia, with 4815/13,083 (36.8%) tweets. In contrast, for paraplegia, neuropathy, and multiple sclerosis, the users who posted the most tweets were “health care professionals and institutions,” with percentages of 43.4% (4305/9920), 67.51% (5131/7600), and 50.54% (7105/14,058), respectively.

However, although “health care professionals and institutions” were the most active users posting about these diseases, public engagement metrics were higher, in terms of likes, for “patients” and, in terms of retweets, for “patient’s acquaintances” (Table 3).

**Table 3.** Count of likes and retweets per tweet classified by different categories: user types, cause, medical tweets, and nonmedical tweets.

Category	Tweets, n (%)		Ratio Likes–tweet, mean (SD)	Ratio retweet-tweet, mean (SD)
Type of user				
Undetermined	11386 (20.53)		128.30 (1868.42)	38.50 (475.56)
Patient	15085 (27.2)		299.97 (2356.87)	44.24 (231.26)
Patient’s acquaintance	4900 (8.84)		258.23 (2616.14)	71.75 (731.90)
Health care professionals or institutions	24080 (43.43)		50.72 (439.10)	20.15 (144.91)
Cause				
Not mentioned	29793 (53.73)		95.91 (1059.18)	28.92 (200.16)
Vaccine or Infectious	3453 (6.23)		191.20 (1512.16)	48.75 (368.00)
Stress	9690 (17.47)		405.75 (3414.54)	64.41 (681.08)
Pharmacological	12515 (22.57)		81.78 (762.54)	23.04 (168.98)
Medical tweets				
Efficacy	20365 (36.73)		98.65 (1209.67)	29.16 (311.48)
Nonefficacy	35086 (63.27)		184.22 (1942.98)	38.46 (360.04)
Nonmedical tweets				
Nonclassifiable/trivialization	8328 (15.02)		431.06 (3301.47)	62.78 (567.84)
Knowledge	23661 (42.67)		59.87 (485.19)	21.53 (146.76)
Commercial	16034 (28.92)		140.20 (1799.59)	37.43 (390.57)
Legal or judicial	7428 (13.4)		164.00 (1335.05)	41.74 (342.92)

### Pharmacological Etiology as the Primary Cause

**Figure 3.**
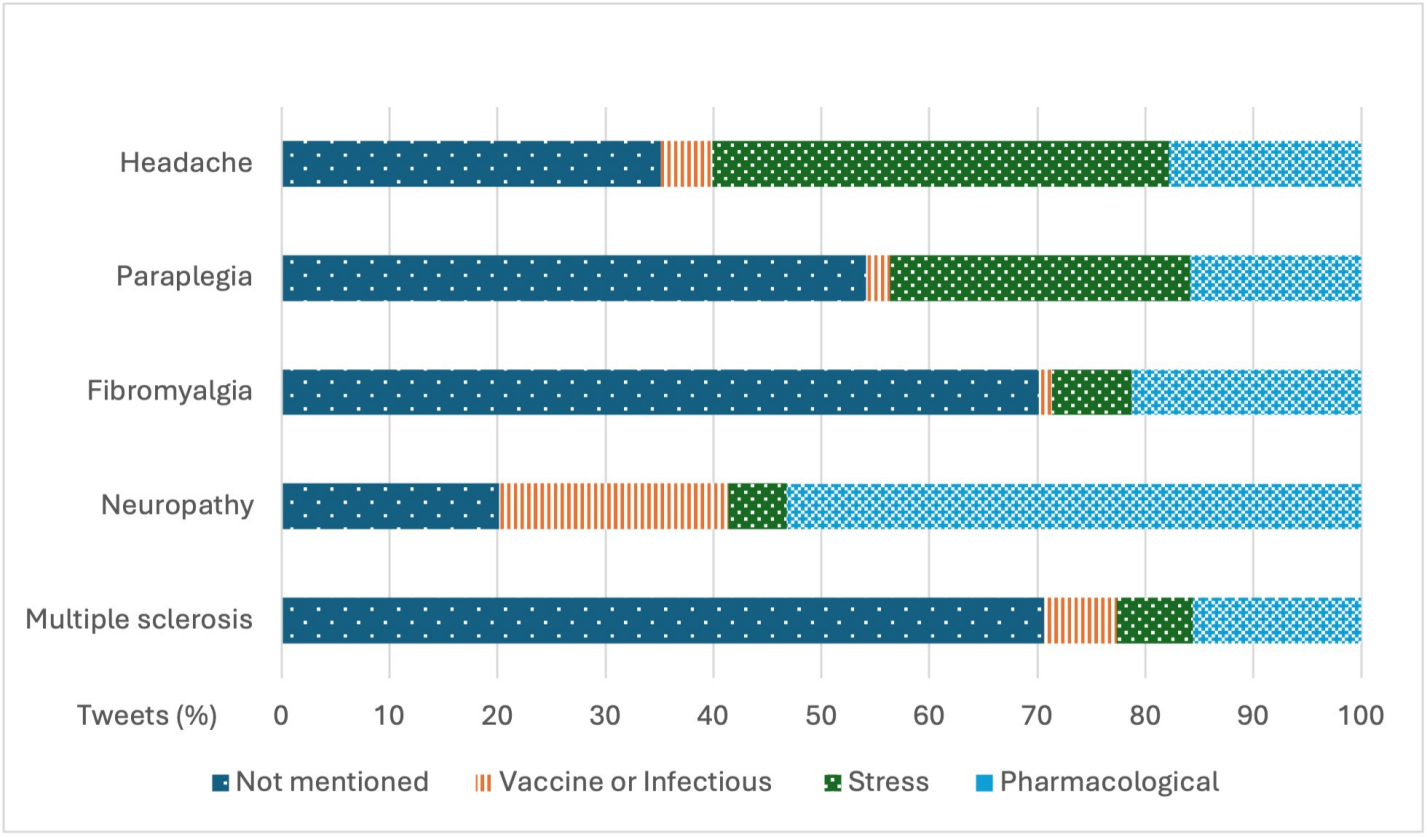
Percentage of tweets related to each etiology by disease.

Out of the 55,451 tweets analyzed, the etiology of the disease was mentioned in almost half of them, with 25,658 (46.27%) tweets. Regarding etiological subcategories, the majority of users identified pharmacological causes as the primary reason for the disease (12,515, 22.57% tweets). Figure 3 shows the distribution of different causes according to the disease. Tweets related to headache and paraplegia more frequently discussed stress-related causes, whereas those related to fibromyalgia, neuropathy, and multiple sclerosis focused more on pharmacological causes.

### Greater Efficacy of Treatments Used for Neuropathy, Paraplegia, and Multiple Sclerosis

**Figure 4.**
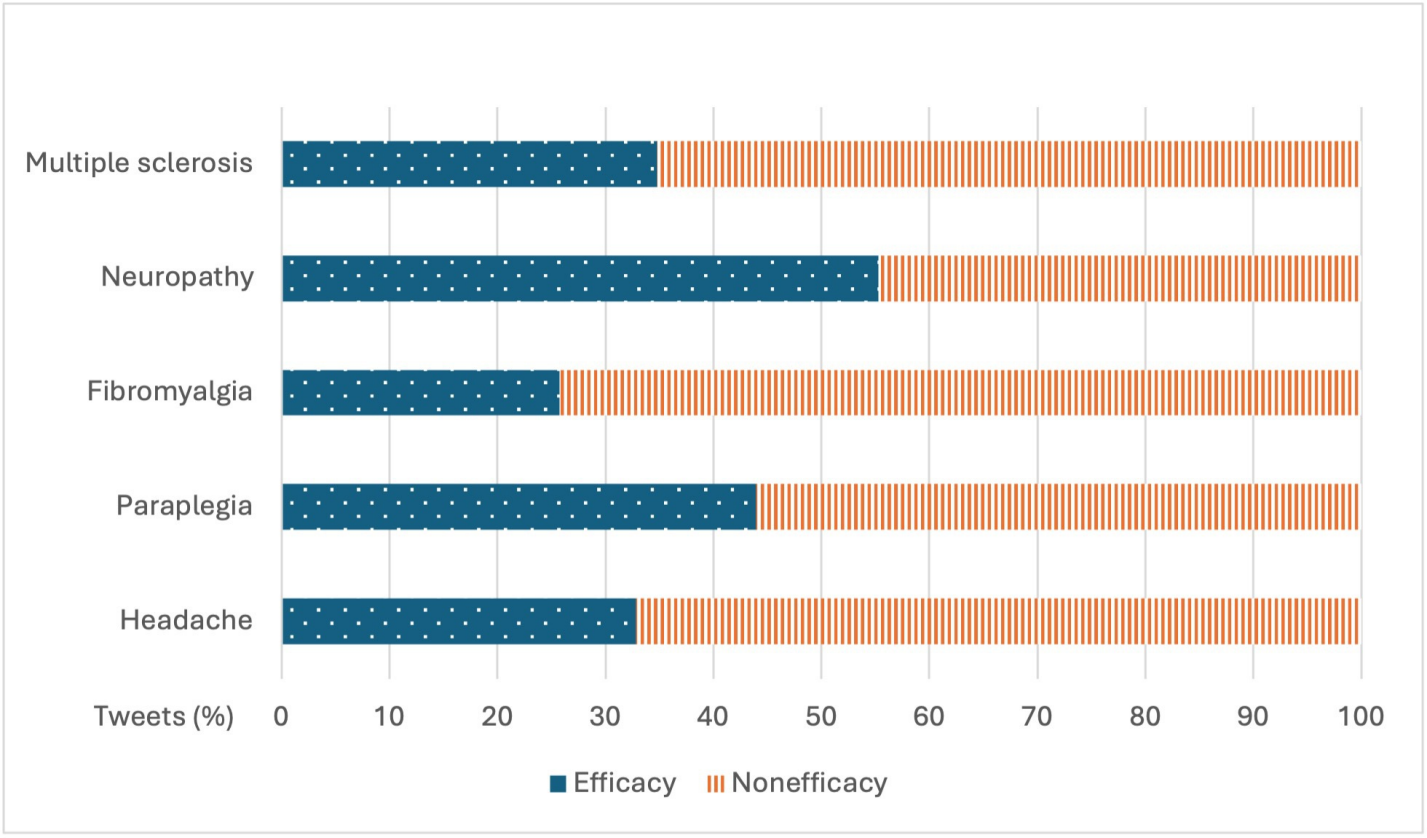
Percentage of tweets related to treatment efficacy by disease.

The analyzed tweets were classified according to their medical and nonmedical content, with “medical” tweets (30,155/55,451, 54.38%) being more prevalent than “non-medical” tweets (25,296/55,451, 45.62%). Within the first group, X users discussed topics, such as the efficacy of treatments used for different diseases. We found differences in users’ perceptions regarding the efficacy of these treatments (Figure 4). On one hand, diseases associated with chronic pain, such as neuropathy, paraplegia, and multiple sclerosis, had a higher percentage of tweets expressing favorable opinions on the efficacy of their treatments, with 55.26% (4200/7600) tweets, 43.96% (4361/9920) tweets, and 34.8% (4892/14,058) tweets, respectively. On the other hand, X users perceived lower efficacy, or did not mention it in the tweet, regarding treatments used for headaches and fibromyalgia, with only 32.81% (3540/10,790) of tweets and 25.77% (3372/13,083) of tweets reporting good efficacy, respectively.

### Tweets Related to Disease Definition and Knowledge Predominated

Regarding content, Table 4 shows the distribution of tweets among the “non-medical” subcategories. The category with the highest proportion of tweets included references to issues related to disease knowledge, totaling 23,661 tweets, which is 42.67% of the analyzed tweets. This was the main area of interest across all the chronic pain–related diseases studied. References to commercial or advertising topics (16,034, 28.92% tweets) and legal issues (7428, 13.39% tweets) were also detected.

**Table 4.** Classification of tweets based on nonmedical content (and their distribution across different subcategories) total and by disease.

Category, n (%)	Total tweets (N)	Headache	Paraplegia	Fibromyalgia	Neuropathy	Multiple sclerosis
Nonclassifiable	8328 (15.02)	3554 (32.94)	1124 (11.33)	1969 (15.05)	791 (10.41)	890 (6.33)
Knowledge	23,661 (42.67)	3164 (29.32)	4596 (46.33)	4701 (35.93)	4541 (59.75)	6659 (47.37)
Commercial	16,034 (28.92)	2764 (25.62)	2727 (27.49)	4140 (31.64)	1296 (17.05)	5107 (36.33)
Legal or Judicial	7428 (13.39)	1308 (12.12)	1473 (14.85)	2273 (17.37)	972 (12.79)	1402 (9.97)

## Discussion

### Principal Findings

Our main findings indicate that multiple sclerosis and fibromyalgia generated the highest volume of tweets while headache generated the highest level of user engagement. The most active users were health care professionals and institutions; however, the tweets that received the greatest reach originated from patients or patients’ acquaintances. Regarding content, the medical perspective predominated, with drug-related etiology being the most frequently mentioned cause by users. A more positive perception of treatment efficacy was observed for multiple sclerosis, neuropathy, and paraplegia, in contrast with a more negative perception in the case of fibromyalgia and headache. Finally, the most frequent nonmedical content was related to general knowledge about the disease.

Our analysis of tweets related to chronic pain conditions, such as headache, paraplegia, fibromyalgia, neuropathy, and multiple sclerosis, follows the model of recent research that uses X to evaluate public interest and communication patterns on health topics [31-36]. The fact that 76.1% of tweets were classifiable suggests that most of the content found was relevant to the study’s objectives. Regarding disease distribution, the 2 dominant topics were multiple sclerosis and fibromyalgia, reflecting greater user participation in conversations related to these conditions. On the one hand, multiple sclerosis, being a chronic and disabling disease, may generate more discussion due to its increasing prevalence in recent years [46,47] and its greater demographic diversification, affecting a wider range of racial and ethnic groups [47,50,55]. In addition, new potential risk factors for disease development have been identified [51], as well as disparities in care and health outcomes among patients with multiple sclerosis based on their socioeconomic status. This relationship is significantly influenced by the high cost of new immunological disease-modifying treatments, which are crucial for managing the disease [52,53]. Therefore, individuals with higher incomes and educational levels tend to experience less disability and less severe symptoms of the disease, even in a context of universal health care access. In contrast, those with lower incomes and educational levels present faster and more severe disease progression [54]. These aspects have contributed to increased public interest and awareness not only among those affected by the disease but also among health care professionals, family members, and researchers.

On the other hand, fibromyalgia also attracts considerable attention due to its controversial nature and diagnostic difficulties, which have led to an increase in public awareness, especially on social media platforms [34]. The like-per-tweet and retweet-per-tweet ratios provide information about the public’s interest in these conversations [43]. In this regard, the disease that has generated the most interest is headache. This is due to its high prevalence, being the most common disease among the 5 studied, affecting up to 9.5% of the global population [56,57], leading X users to actively seek support and empathy on social media platforms [58].

Our results also show that individuals with lived experience and their acquaintances appear to be less active in X discussions about chronic pain conditions, while health care professionals and institutions are the most active users, with their content focusing primarily on medical aspects. We expected that individuals with lived experience would be more willing to discuss these diseases, as observed in previous studies [31,32]. The significant presence of health care professionals on X is a positive finding. The use of social media by health care professionals facilitates the dissemination of health-related information and promotes 2-way communication with users [35,36]. However, it is “patients” who speak about their own experiences related to headaches and fibromyalgia. A speculative explanation is that these diseases have a worse social perception compared to other conditions due to the invisibility and subjectivity of their symptoms. The emotional and psychological burden of living with these invisible conditions often leads patients to seek validation and social support. Therefore, these platforms may allow patients to express their frustrations and seek advice from others experiencing similar pain and symptoms [34,59]. These lived experiences could help reduce feelings of invalidation and better tailor therapeutic efforts [60].

Recent research highlights how digital platforms are valuable tools for assessing public perception, understood as the overall perspective expressed by patients, acquaintances, institutions, and other users, regarding the etiology of diseases [61-63]. First, the perception that stress is related to the onset and exacerbation of symptoms in conditions, such as headaches and paraplegia, is supported by the scientific literature. Several studies have found that stress is the main trigger for migraine episodes in a large proportion of patients [63-65], while in the case of paraplegia, although the primary cause is usually physical damage to the spinal cord, stress plays an important role in exacerbating symptoms, such as chronic pain and muscle tension. Studies have shown that individuals with spinal cord injuries may experience increased pain in situations of high stress [66-68].

Second, X users provide insights into how drugs may be implicated in the development or exacerbation of fibromyalgia, neuropathy, and multiple sclerosis. This claim aligns with a review of studies exploring how medications can influence these diseases. For example, in the case of fibromyalgia, the authors discuss how certain medications can cause similar symptoms, as well as induce or exacerbate disease symptoms [69,70]. Several papers also analyze how different drugs can induce neuropathies and review the mechanisms behind nerve damage caused by these medications [71,72]. Finally, a review discusses drug-induced multiple sclerosis-like syndrome and explores the influence of pharmacological treatments on the induction, progression, and severity of the disease [73]. Therefore, all this justifies the increased appearance of these etiological subcategories in user discussions on platforms like X. In addition, we are concerned about how poor treatment adherence for diseases, such as fibromyalgia, neuropathy, and multiple sclerosis, may be influenced by these beliefs about medication and concerns about their long-term adverse effects [74-76].

The analyzed data generally show that “medical” topics were more frequent than “non-medical” ones. Regarding “medical” content, X users discuss the effectiveness of treatments used for the studied diseases to seek support from others going through similar situations and exchange information and experiences about different therapies [77]. In this way, our research improves knowledge about public opinions, for example, on emerging therapies. Notable therapeutic innovations include the use of monoclonal antibodies targeting the calcitonin gene-related peptide, which have shown efficacy in preventing chronic migraines [78]. In patients with spinal cord injuries causing paraplegia, options such as spinal cord stimulation are being explored for pain treatment, with promising approaches enhancing neural plasticity [79-81]. In fibromyalgia, advances in understanding its underlying mechanisms have enabled research into repetitive transcranial magnetic stimulation [79,82] and cannabinoid therapy as potential treatments to relieve pain in these patients [83,84]. Cannabinoids and magnetic stimulation [84], as well as nanomedicine-based therapies [79], are also being explored as future options in neuropathy; the frequent debilitating chronic pain in multiple sclerosis progression has also required the exploration of treatments with cannabinoids [85] and biologic therapies aimed at modulating the immune system and reducing inflammation [86].

Neuropathy and multiple sclerosis, despite being difficult-to-treat conditions, have therapeutic options that are appreciated by patients because they provide significant pain relief and improve quality of life. Advances in treatments for multiple sclerosis and paraplegia have also generated expectations and positive experiences, as reflected on social media [87,88]. Notably, despite the lack of a cure for multiple sclerosis and paraplegia, X users post about the effectiveness of their treatments because they value the improvement in quality of life, personalized therapeutic advancements, and the psychological and social support they receive [81,89-93]. In addition, continuous innovation in research generates hope, reinforcing this positive perception due to the possibility of maintaining independence and functionality for a longer time [81,87]. However, current treatments for headache and fibromyalgia do not always achieve significant relief, increasing dependence on medications and their side effects [6,94-98]. The limited therapeutic efficacy, the invisibility of symptoms, and the frequent lack of both social and medical understanding generate frustration among patients [26,28,99], which may be reflected in social media data [47], as observed in our study, and helps contextualize differences in treatment perception.

The codebook also revealed a noteworthy theme related to the proportion of information and knowledge about diseases. It has been shown that inadequate knowledge of a specific disease can lead to the stigmatization and discrimination of patients [100]. An example is the historical rejection faced by patients with epilepsy. It was often believed that epilepsy had its origins in malevolent causes or was associated with sin or demonic possession, as well as the theory of epilepsy as contagion and madness [101-105]. These phenomena arise from misunderstandings or insufficient knowledge about why epileptic seizures occur [106]. Similarly, the stigmatization of depression is deeply rooted in the lack of information and understanding about the disease [107,108]. Therefore, chronic pain, which is difficult to quantify and measure, is often minimized or ignored by professionals and society, contributing to a perception of exaggeration or invalidation of the patient’s experience. Among the conditions studied, headache and fibromyalgia are the least understood, due to the invisibility of their symptoms, the lack of objective biomarkers, and diagnostic ambiguity [109-111]. Patients often perceive this invalidation from family members, the health care system, and society at large [112,113], which may exacerbate symptoms and negatively impact their emotional well-being [60,113-115].

Studies have demonstrated that when a disease and its treatment options are better understood, patients are more likely to follow medical recommendations and have higher adherence rates [116-119]. However, current treatment options for headache and fibromyalgia exhibit limited efficacy, focusing primarily on symptomatic relief and often being associated with adverse effects [41,42,94-98]. These therapeutic limitations, combined with the multifactorial complexity of these conditions and the perceived lack of validation, contribute to patient frustration [40-42] and diminished trust in the health care system. Such experiences are frequently expressed on social media [34, 41], providing valuable insights into public perceptions of treatment effectiveness. In this context, social media platforms serve a dual role: they facilitate the sharing of emotional and personal experiences and function as channels for health education. The active engagement of health care professionals and institutions on these platforms can help counteract misinformation and provide trustworthy resources, particularly in digital environments where false information spreads rapidly [120,121]. Moreover, these interactions promote more open communication between patients and health care providers, supporting stigma-reduction efforts and enhancing awareness-raising strategies.

Overall, the findings of this study offer practical implications for public health, health communication, and clinical practice. They demonstrate how social media content analysis can complement traditional research, capture real-time social perceptions, and inform more effective education and awareness strategies.

### Limitations

When interpreting the findings of this study, several limitations must be considered. Although our search tool accessed 100% of available tweets, some mentions of chronic pain conditions may have been missed due to the use of alternative keywords. The presence of abbreviations, grammatical errors, and colloquial language by users may have hindered the accuracy of data retrieval and analysis. In addition, not all chronic pain conditions were included. The demographic profile of X users, generally younger individuals with specific socioeconomic characteristics, does not reflect the general population, which may limit the generalizability of our findings to broader discussions on chronic pain. As with most qualitative research, the development of the codebook and tweet analysis involves a degree of subjectivity. Although predefined criteria were applied, the analysis was conducted by a single coder, which may have influenced the interpretation of certain messages. Moreover, tweet content may evolve over time, and the inclusion of only English and Spanish tweets could distort the perception of some health-related issues. The potential influence of bots and fake accounts may have affected the data to some extent; however, specific tools were applied to detect or exclude such accounts. Likewise, interactions on X, such as retweets and likes, do not necessarily reflect users’ true understanding or perceptions of a topic, but rather the content’s popularity or immediate appeal, as well as the influence of accounts with large followings. Finally, our methodological design does not allow for an in-depth exploration of direct relationships between social media use and specific clinical outcomes due to the anonymity of the data obtained.

Nevertheless, this study used a methodology that has been consistently used in previous medical research on X [44-47,50-53], and it offers a valuable approach to exploring the dynamics of social media discourse related to chronic pain conditions.

### Conclusions

Our study addresses key aspects related to chronic pain and its implications for public health. It is observed that the main participants in discussions about these conditions are health care professionals and institutions, highlighting an opportunity to improve the dissemination of accurate information and optimize the understanding and treatment of this condition. Social media platforms, such as X, play a crucial role as key resources for discussions about chronic pain among health care professionals and patients. Furthermore, the predominance of discussions focused on treatment reflects the influence of available therapeutic options and their perceived effectiveness. Finally, the study underscores the need to enhance education and public awareness, as a significant portion of the content on social networks centers around the definition and understanding of chronic pain from a nonmedical perspective. This highlights the potential of social media to correct misconceptions and provide better support to patients.

## Supplementary material

10.2196/67473 Multimedia Appendix 1 Supplementary codebook.
