# Supplementary material for: Exploring Pain on Social Media: Observational Study on Perceptions and Discussions of Chronic Pain Conditions
Source: JMIR Infodemiology. 2025 Sep 16;5:e67473. doi: 10.2196/67473 (PMC12440231; doi:10.2196/67473)
Supplement: Multimedia Appendix 1 [file infodemiology-v5-e67473-s001.pdf]

CODEBOOK

DISEASE: *(and their equivalents in Spanish)*

1. Headache, Migraine (1)
2. Paraplegia, Tetraplegia (2)
3. Fibromyalgia (3)
4. Neuropathy, Polyneuropathy (4)
5. Multiple Sclerosis (5)

A. Whether it is classifiable (1) or not (0):

0. **NOT CLASSIFIABLE:** *These are cases in which the language used differs from English or Spanish, or when the information provided is not sufficiently clear or developed to understand the tweet's meaning.* → **No further analysis is conducted**.

1. **CLASSIFIABLE:** *All others: English or Spanish. The tweet provides relevant information. If the tweet is classifiable →* **the following points are analyzed**:

- **USER:** *Refers to the individual or organization that posts or shares the tweet. It is coded according to the user profile, the tweet content, and the use of pronouns*.

1. **Patient (1):** *The user refers to their own experience with the disease. Identified using the first person or direct mention of their condition*.
2. **Relative or acquaintance of the patient (family member, friend, close person) (2):** *The user refers to the experience of a family member or friend with the disease. Identified using the third person (e.g., "my mother").*
3. **Healthcare professionals or healthcare/pharmaceutical institutions (hospitals, universities, scientific societies, associations, research groups, scientific journals, and pharmaceutical industries) (3):** *The user is recognized as a healthcare professional (doctor, therapist, etc.) or as an institutional account (hospital, healthcare NGO, medical society, etc.). The user is identified by the profile, credentials, user description, or shared content.*

- **CONTENT ANALYSIS:** MEDICAL CONTENT (1) / NON-MEDICAL CONTENT (2). *Choose the predominant one; they are not mutually exclusive*.

1. **MEDICAL CONTENT (1):** *Refers to information directly related to clinical, etiological, or therapeutic aspects of the disease.*

- **ORIGIN/CAUSE:** *Refers to the likely etiology of the disease. It is coded according to explicit content.*

1. **Previous vaccination or infection (1):** *Previous infections or post-vaccine processes are mentioned.*
2. **Stress (2):** R*eference is made to physical or emotional stress as a trigger for the disease.*
3. **Drugs (3):** *Medications are mentioned as the cause of the disease*.

- **TREATMENT EFFICACY:** *Refers to the perception expressed in the tweet about the effectiveness of a treatment for chronic pain associated with the disease in question.*

1. **Effective for chronic pain (1):** *The treatment is effective or beneficial for the chronic pain of the disease.*
2. **Not effective for chronic pain (2):** *The treatment is not effective or beneficial for the chronic pain of the disease.*

2. **NON-MEDICAL CONTENT (2):** *Includes topics not focused on clinical, etiological, or therapeutic information, though they are related to the disease from other perspectives.*

1. **Knowledge (1):** Refers to general information about the diseases: definitions, theories, criteria, classifications, etc.
2. **Commercial/Advertising (2):** Promotion of events, products, or fundraising related to the disease or similar topics.
3. **Legal/Judicial (3):** Refers to complaints or claims in political, social, or legal contexts.
